# Investigation of the effects of pretreatment on the elemental composition of ash derived from selected Nigerian lignocellulosic biomass

**DOI:** 10.1038/s41598-021-00672-1

**Published:** 2021-10-29

**Authors:** Adeolu A. Awoyale, David Lokhat, Patrick Okete

**Affiliations:** 1 Reactor Technology Research Group, School of Engineering, University of KwaZulu-Natal, Durban, South Africa; 2 Petroleum and Natural Gas Processing Department, Petroleum Training Institute, Effurun, Nigeria

**Keywords:** Energy science and technology, Engineering

## Abstract

Lignocellulosic biomass is an important source of renewable energy and a potential replacement for fossil fuels. In this work, the X-ray fluorescence (XRF) method was used to analyze the elemental composition of raw and pretreated lignocellulosic biomass of cassava peels, corn cobs, rice husks, sugarcane bagasse, yam peels, and mixtures of cassava peels and yam peels, corn cobs and rice husks and all five biomass samples combined. The influence of particle size on elemental properties was investigated by screening the selected biomass into two size fractions, of an average of 300 and 435 µm, respectively. The total concentration of Mg, Al, Si, P, S, Cl, Ca, Ti, Cr, Mn, Fe, Co, Cu, Zn, Sn, Ni, Br, Mo, Ba, Hg, and Pb were determined for each of the biomass samples before and after the different pretreatments adopted in this study. From the results of the analysis, there was a significant reduction in the concentration of calcium in all the analyzed biomass after the alkaline pretreatment with rice husks biomass having the lowest concentration of 66 ppm after the alkaline pretreatment. The sulfur content of the acid pretreated biomass increased considerably which is likely due to the sulfuric acid used for the acid pretreatment. The fact that a mixture of biomass feedstock affects the properties of the biomass after pretreatment was validated in the mixed biomass of cassava peels and yam peels biomass as an example. The concentration of Mg in the mixed biomass was 1441 ppm but was 200 ppm and 353 ppm in individual cassava peels and yam peels respectively. The results of this study demonstrated that pretreated mixtures of biomass have varied elemental compositions, which could be an important factor affecting downstream processes, especially if a hybrid feedstock is used in a large-scale application.

## Introduction

The use of fossil fuels (petroleum, coal, and natural gas) from decomposing plants and animal remains for energy generation has posed several challenges to the environment due to their non-renewability nature and their adverse depletion of the ozone layer due to the release of greenhouse gases (GHG)^1^. The negative impacts of the use of fossil fuels on the environment have led to the introduction of biomass as a renewable and environmentally friendly fuel source. Biomass is a complex heterogeneous mixture of organic materials, inorganic materials, containing various solid and fluid, intimately associated minerals of different sources^2^. The US patent on processing biomass defined biomass as any non-fossilized organic matter^3,4^. They include cellulosic and lignocellulosic materials such as plant biomass, animal biomass, and municipal waste biomass^5^. They also include HAR-herbaceous and agricultural residues: shells, cobs, and husks of plants and others^6^. Sanderson^7^, defined lignocellulosic biomass as the inedible parts of plants that are feedstocks for the next generation of biofuels. Technically, lignocellulosic materials are a mixture of natural polymers (carbohydrates) such as lignin, cellulose, and hemicellulose, and tannins with more than two hydroxyl groups per molecule. Waste biomass is produced as a low value by-product of various industrial sectors such as agriculture. It includes corn stover, sugarcane bagasse, straws, sawmill, and paper mill discards^8^.

Typical lignocellulosic biomass contains 30–50% of cellulose, 15–35% of hemicellulose, and 10–30% of lignin^9^. With the complex nature of lignocellulosic materials, it poses resistance to chemical and biological degradation during enzymatic hydrolysis and subsequent fermentation. This highly recalcitrant nature of lignocellulosic materials in the production of biofuels, mainly ethanol makes the process economically unfeasible and thus, pretreatment is needed before saccharification and fermentation^10,11^. The constituents of lignocellulosic biomass also include inorganic matters present in a trace concentration and are essential for plant growth. The inorganic constituents of biomass consist of macronutrients and micronutrients. The macronutrients are nutrients needed by plants in a very large concentration. They include nitrogen (N), potassium (K), magnesium (Mg), phosphorus (P), calcium (Ca), and sulfur (S). Micronutrients on the other hand are needed by plants in relatively small concentration. Examples of such micronutrients are copper (Cu), manganese (Mn), zinc (Zn), iron (Fe), boron (B), molybdenum (Mo), and chlorine (Cl). Other minerals that are of great benefit to plants but are not essential are sodium (Na), vanadium (V), nickel (Ni), cobalt (Co), aluminum (Al), silicon (Si), and selenium (Se). The elements function differently in plants, from the production of amino acids, protein synthesis, enzyme activation, nucleic acids, energy household, ATP, cell wall structure to photosynthesis reaction, among others.

In time past, the utilization of lignocellulosic biomass was restricted to combustion for domestic and industrial heating with attendant adverse effects on the environment. Problems such as land degradation and desertification have been associated with the use of lignocellulosic biomass. In recent times, researchers have come up with a better and more economically viable means of lignocellulosic biomass utilization with a minimal negative impact on the environment. Lignocellulosic biomass conversion routes such as thermochemical or biochemical processing methods afford their conversion to energy or energy carriers. The thermochemical processing route employs heat and chemical means such as combustion, pyrolysis, gasification, and liquefaction in the production of energy products from lignocellulosic biomass whereas the biochemical processing route adopts the use of microorganisms or enzymes and bacteria in the decomposition of the biomass to obtain biofuels^12^. The thermochemical processes normally require a large amount of energy, as well as the inclusion of a solvent or catalyst. The biochemical approach has a longer cycle time and is less effective at breaking down resistant biomass components. By integrating the advantages of both ways in biofuel manufacturing, combining the two routes can be promising. When hydrothermal routes are utilized in the pretreatment stage to prepare the suitable biomass feedstock for the subsequent biological routes, the total process efficiency and final product yields are improved, and vice versa^13^.

Depending on the various forces or resources utilized in the pretreatment process, pretreatment technologies can be classified as physical, chemical, biological, or physio-chemical, or a mix of these.

Liquid hot water (LHW) pretreatment is a type of hydrothermal pretreatment that does not require fast decompression or the addition of a catalyst or chemical. LHW pretreatment uses a temperature and pressure range of 170 to 230 °C and > 5 Mpa, respectively. The LHW pretreatment removes hemicellulose from lignocellulosic materials, exposing the cellulose and allowing fermentation to take place^14^.

Acid pretreatment is a type of chemical pretreatment involving chemical hydrolyses that solubilize hemicellulose and lignin, allowing enzymes to work on the cellulose during the fermentation process. Acid pretreatment can be done with concentrated or dilute acid, however, the use of concentrated acid has the drawback of the formation of inhibiting compounds such as furfural and phenolic acids; also, concentrated acids are unpleasant, caustic, and generally dangerous. As a result, corrosion-resistant equipment should be used for this pretreatment process. For large-scale bioethanol production, dilute acid pretreatment is the best option.

An alkaline pretreatment is a type of chemical pretreatment in which the biomass is treated with a base such as potassium, or calcium hydroxides at standard pressure and temperature. This pretreatment process offers the advantage of eliminating lignin from the biomass more effectively. The technique also removes acetyl and uronic acid groups from hemicellulose, increasing the enzyme's accessibility to break down hemicellulose Pretreatment with alkali can also be done at low temperatures, pressures, and times^15^.

Shen et al.^16^ worked on the prediction of the elemental composition of biomass based on proximate analysis. New correlations for determining elemental composition based on the proximate study of biomass was presented in the research work. The established correlations can be utilized for the accurate computation of elemental composition of different biomass particularly for biomass with high ash content, after the proximate analysis.

Yusuf et al.^17^ worked on the characterization of Ugandan biomass wastes as the potential candidates for bioenergy production. In the work, various analyses were performed on Mbwazirume peel (MP) and Nakyinyika peel (NP) biomass, including proximal and ultimate such as TGA, FT-IR, AAS, and SEM–EDS. The result of analysis shows that the components identified in both ash deposits were sorted as follows during the EDS analysis: For MP, O > K > C > Cl > Mg > P, while for NP, K > Cl > Mg > P > Al.

Osman et al.^18^ investigated the physiochemical characterization of miscanthus and its application in heavy metals removal from wastewaters. A novel alternative use was studied in this study, namely the direct use of dried miscanthus (DM) plant as an adsorbent for heavy metals removal (HMR) from wastewaters. XRD, S_BET_, TGA, DSC, SEM–EDX, elemental analysis, halogen, and ICP techniques were used to investigate the physical, chemical, and leaching properties of DM. The results show that the DM sample had 42.85% carbon, 5.83 percent hydrogen, 1.21 percent nitrogen, 0.1 percent sulfur, and 50.01 percent oxygen.

The aim of this study is therefore to determine the effects of three pretreatment methods on the elemental composition of ash materials in selected Nigerian lignocellulosic biomass individually and as hybridized (mixed) feedstocks in bioethanol production. Knowledge of the ash content helps to estimate the possibility of slagging and scale formation in the process of combusting or gasification of biomass while the estimation of the elemental composition helps determine the conversion efficiency of the biomass in bioethanol production. Also, a hybridized (mixed) biomass feedstocks help to guarantee enough feedstock for large-scale bioethanol production plants, hence information on the mixed feedstocks would aid in equipment design and selection of process conditions/methods.

## Experimental procedures/methods

### Biomass preparation

The corncobs were obtained from Ogume, a village in Ndokwa West Local government area of Delta State, Nigeria. The rice husk used for this work was obtained from a rice mill in Ekperi in Etsako Central Local Government area of Edo State, Nigeria. The other biomass, yam peels, cassava peels, and sugarcane bagasse were locally sourced within Effurun in the Uvwie Local Government Area of Delta State, Nigeria. All biomass were sundried for about 7 days and then taken to the mill where they were ground into a powder. After grinding, the ground biomass were sieved into particle sizes of 300 μm and 425 μm. Pretreatment of biomass was carried out using analytical grade chemical reagents such as sodium hydroxide pellets, hydrogen peroxide, and tetraoxosulphate (VI) acid.

1500 g of each biomass was collected into different containers except for sugarcane bagasse for which 1000 g was collected. To verify the effect of biomass combination on the elemental composition, 300 g each of 300 μm sized particles for all five biomass samples were collected into another container and 750 g of 300 μm sized particles of cassava peels and yam peels combined and corn cobs and rice husks combined were measured into two containers respectively. Thorough mixing of the combined mixture of two or more biomass was ensured for uniformity in composition. Details of the pretreatment process can be obtained from previous work by the authors^15^.

### Ash content

The procedure depicted in ASTM D2017 (1998) was used in determining the ash content of the biomass samples in this study. 1 g of sample was placed in a pre-weighed crucible and incinerated in a muffle furnace at 760 °C until complete ashing was achieved. The crucible was then transferred into a desiccator for cooling. Three replicates were made. The cooled samples were then weighed. The ash content was calculated using the equation below.

1 $$ {\text{Ash}}\,{\text{Content }}\left( \% \right) = \frac{{{\text{weight}}\,{\text{of}}\,{\text{ash}}}}{{{\text{original}}\,{\text{weight}}\,{\text{of}}\,{\text{sample}}}} \times 100 $$

### Xrf analysis

X-ray fluorescence (XRF) spectroscopy is a rapid method used to determine the biomass ash composition. The XRF provides simple analytical solutions to a wide range of quality and process control requirements when compared to other analytical techniques. It can provide detailed analysis on non-destructive analysis, minimal preparation samples, simultaneous multi-element quantitative and qualitative analysis and these results are displayed in seconds. In this study, X-ray fluorescence (x-supreme 8000), incorporated with an application-optimized Oxford instrument’s high-reliability X-ray tube and high-performance silicon drift (SDD) was used to analyze the elemental composition of the ashes obtained from the selected lignocellulosic biomass being studied. To minimize the error, multiple experiments (triplicate) were carried out for each sample, and the average value was selected for the chemical composition of the samples. XRF analysis was carried out on the ash obtained from the different biomass selected for this study. The elemental composition in 1 kg of the different biomass and biomass mixtures was given in ppm.

## Results

### General observations

From the result of the experiment, it was seen that the chemical composition of the mineral matter of ashes depends largely on the biomass type, origin, and combustion conditions as corroborated by previous researches on ash components and the varying composition of ash in comparison to the combustion temperature. The lignocellulosic biomass used in this study has varying ash chemical composition for cassava peel, corn cobs, rice husk, sugarcane bagasse, and yam peels as shown in Table 1. The main inorganic mineral constituents of the biomass are Ca, Fe, S, Si, Al, P, and Sn. The five major inorganic constituents of the biomass are shown in decreasing order of abundance as shown in Table 2. The concentration of the elements in the raw biomass samples follows the same trend as that obtained by Cavalaglio et al.^19^ in their work centered on the Characterization of Various Biomass Feedstock Suitable for Small-Scale Energy Plants as Preliminary Activity of Biocheaper Project.

**Table 1:** Effect of acid, alkaline, and hydrothermal pretreatment on the raw biomass samples.

Element	Mg	Al	Si	P	S	Cl	Ca	Ti	Cr	Mn	Fe	Co	Cu	Zn	Sn
**Cassava peels (ppm)**															
R	55	175	630	117	287	47	2014	284	9	19	1074	0	0	18	0
A	354	1111	4014	239	14,044	18	1805	454	9	15	654	0	0	9	523
B	0	436	821	58	223	40	599	100	0	0	383	0	0	14	0
C	393	664	1104	219	333	57	1136	176	0	11	628	0	0	9	0
Mean	201	597	1642	158	3722	40	1389	253	4	11	684	0	0	12	131
**Corn cobs (ppm)**															
R	712	344	416	535	374	487	135	53	0	11	144	0	0	13	803
A	0	76	351	142	429	198	1242	146	0	10	317	0	0	8	0
B	41	137	194	43	81	85	84	41	0	0	124	0	0	14	1105
C	142	323	468	160	222	140	135	55	6	8	161	0	0	16	536
Mean	224	220	357	220	276	228	399	74	1	7	187	0	0	13	611
**Rice husks (ppm)**															
R	511	324	1299	431	5083	33	68	29	0	18	36	0	0	0	0
A	774	490	4070	985	7517	14	65	58	0	28	72	0	0	9	0
B	53	28	376	356	54	30	66	34	0	45	81	0	0	18	430
C	1461	134	2303	1986	939	0	170	58	0	103	148	0	0	32	613
Mean	700	244	2012	940	3398	19	92	45	0	48	84	0	0	15	261
**Sugarcane bagasse (ppm)**															
R	0	194	824	332	348	250	901	123	0	17	1368	0	10	14	612
A	0	796	1425	311	16,197	15	382	128	0	98	955	0	34	15	0
B	0	1180	292	35	115	48	128	39	0	24	131	0	0	9	0
C	0	92	648	220	325	179	1009	121	0	20	1141	0	14	15	376
Mean	0	565	797	224	4246	123	605	103	0	40	944	0	15	13	247
**Yam peels (ppm)**															
R	0	331	631	334	263	74	272	589	0	61.5	1213	0	0	8	1516
A	287	1235	3574	353	7641	17	217	1085	0	33	1170	0	0	7	0
B	92	265	534	374	385	307	168	132	0	12	201	0	0	9	1364
C	1032	780	1356	1154	434	120	302	463	0	68	1411	11	0	15	0
Mean	353	653	1524	554	2181	130	240	567	0	44	999	3	0	10	720

R = Raw.

A = Acid pretreated.

B = Alkaline Pretreated.

C = Hydrothermal Pretreated.

**Table 2:** Major inorganic constituents of the lignocellulosic biomass in this research.

Biomass (Raw Samples)	Elemental composition (decreasing order of abundance)
Cassava peels	S > Si > Ca > Fe > Al
Corn cobs	Sn > Ca > Si > S > Cl
Rice husks	S > Si > P > Mg > Sn
Sugarcane bagasse	S > Fe > Si > Ca > Al
Yam peels	S > Si > Fe > Sn > Al

### Effects of particle size

Particle size affected the elemental composition of the raw biomass being studied. This could be as a result of the presence of mineral matters of technogenic origin that are present in the biomass particles that were not broken to the particle size of ≤ 300 μm sizes or the presence of these minerals in 300 μm sized particles and absent in this same proportion in 425 μm sized particles. For example, from Table 3, Sn is present in the 425 micron-sized cassava particles but absent in the 300 m sized cassava particles. Also, Cr, a trace element, was present in the 300 micron-sized cassava peel biomass particles but absent in the 425 micron-sized particles. Sn, a major constituent of unpretreated or raw 300 micron-sized corn cobs, was absent in the 425 m size. This explains why varying particle sizes may also vary the inorganic mineral constituents or composition (ash content) of the biomass as corroborated by Lori et al.^20^ in their work on proximate and ultimate analyses of bagasse, sorghum and millet*.* Cassava peel biomass is typically rich in Ca with a 2014 ppm and 2098 ppm mass for 300 and 425 μm sized particles respectively. This kind of variation in the concentration of the elemental composition is observed all through the biomass considered in this research. The result also shows that magnesium has a higher concentration in the 425 microns sized biomass for all the biomass being studied except for corn cobs biomass where its concentration is higher in the 300 microns sized biomass. The concentration of zinc in all the biomass in this study is low and the effect of particle size on it is not too pronounced.

**Table 3:** Effects of particle size.

P.S*(μm)	Mg	Al	Si	P	S	Cl	Ca	Ti	Cr	Mn	Fe	Co	Cu	Zn	Sn
**Cassava peels (ppm)**															
300	55	175	630	117	287	47	2014	284	9	19	1074	0	0	18	0
425	753	851	521	244	431	42	2098	139	0	21	433	0	0	10	1028
**Corn cobs (ppm)**															
300	712	344	416	535	374	487	135	53	0	11	144	0	0	13	803
425	169	162	355	253	225	398	36	38	3	7	58	0	0	12	0
**Rice husks (ppm)**															
300	511	324	1299	431	5083	33	68	29	0	18	36	0	0	0	0
425	5306	347	4605	3331	383	20	146	53	0	81	91	0	0	17	0
**Sugarcane bagasse (ppm)**															
300	0	194	824	332	348	250	901	123	0	17	1368	0	10	14	612
425	204	311	644	341	353	207	690	66	0	10	548	0	0	9	397
**Yam peels (ppm)**															
300	0	331	631	334	263	74	272	589	0	61.5	1213	0	0	8	1516
425	1117	933	656	788	592	29	507	277	6	73	866	4	5	10	0

P.S*^—^Particle size.

### Effects of pretreatment

The chemical pretreatment employed in this study was a determinant factor in the elemental composition of the ash content in the biomass being studied. The analysis of the effects of pretreatment on the elemental composition was focused on the 300 μm sized particles. The alkaline pretreatment was seen to reduce the Ca concentration in all the biomass tremendously. The acid pretreatment was seen to increase the sulfur content of the biomass. This is due to the presence of sulfur in the acid (H_2_SO_4_) used for the pretreatment. High concentrations of Si and Ca form low—melting–point eutectics, which can cause slagging. Salts of these elements do form surface deposits on heating equipment^21^. The five-biomass mixture has a relative concentration of chlorine with maximum concentration in corn cob and sugarcane bagasse with values of 487 ppm and 250 ppm respectively. Chlorine is a major parameter in ash deposits. Its presence reduces the melting point of ash and therefore allows for an easier deposition of ash. Al compounds also play a key role in reducing the melting point of ash. Sulfur oxides form sulphates and condense on the surface of heating equipment. They also form fly ash particles. Generally, fuels with high Ca content will have higher sulfur fixation in the ash^20,22^. Ca and Mg in a biomass fuel increase the ash melting point temperature of the fuel, thus making it more suitable for power plant fuel as against the high concentration of potassium which will, in turn, lead to slagging and formation of hard deposit in the furnace and reboiler. The high phosphorus content of rice husks in hydrothermal pretreatment will influence the burning properties as well as cause the formation of low melting temperature ash^23^.

Elements often associated with environmental toxicity are present in the biomass in very minute concentrations. Heavy elements such as Co and Cu were present only in sugarcane bagasse and yam peels and absent for all other samples. It was also noticed that Co and Cu which were present in raw sugarcane bagasse and yam peels were absent after both samples underwent alkaline pretreatment. The impact of alkaline pretreatment was evident in Mg and most of the biomass samples, reducing to 0.000 ppm or very low values. Alkaline pretreatment could be said to be relevant in removing some toxic minerals present in the biomass. Other toxic substances in the biomass include Al, Mn, Cr, and Zn but in trace concentration. It is important to also note that the presence of these substances in the given samples of sugarcane bagasse and yam peels does not validate their presence in all samples of yam peels and sugarcane bagasse as the elemental composition varies with growth processes, growing conditions (such as sunlight, geographical location, climate, seasons), fertilizer and pesticides doses, plant distance from the source of pollution (plant environment), harvesting time, blending of different biomass types, and others^2,24^. Sugarcane bagasse and rice husks which are characterized as herbaceous and agricultural residues (HAR)^2,6^ contained the highest concentration of silicon with values of 824 ppm and 1299 ppm respectively. Mg though present in all other samples was absent in sugarcane bagasse.

#### Acid pretreatment

**Figure 1:**
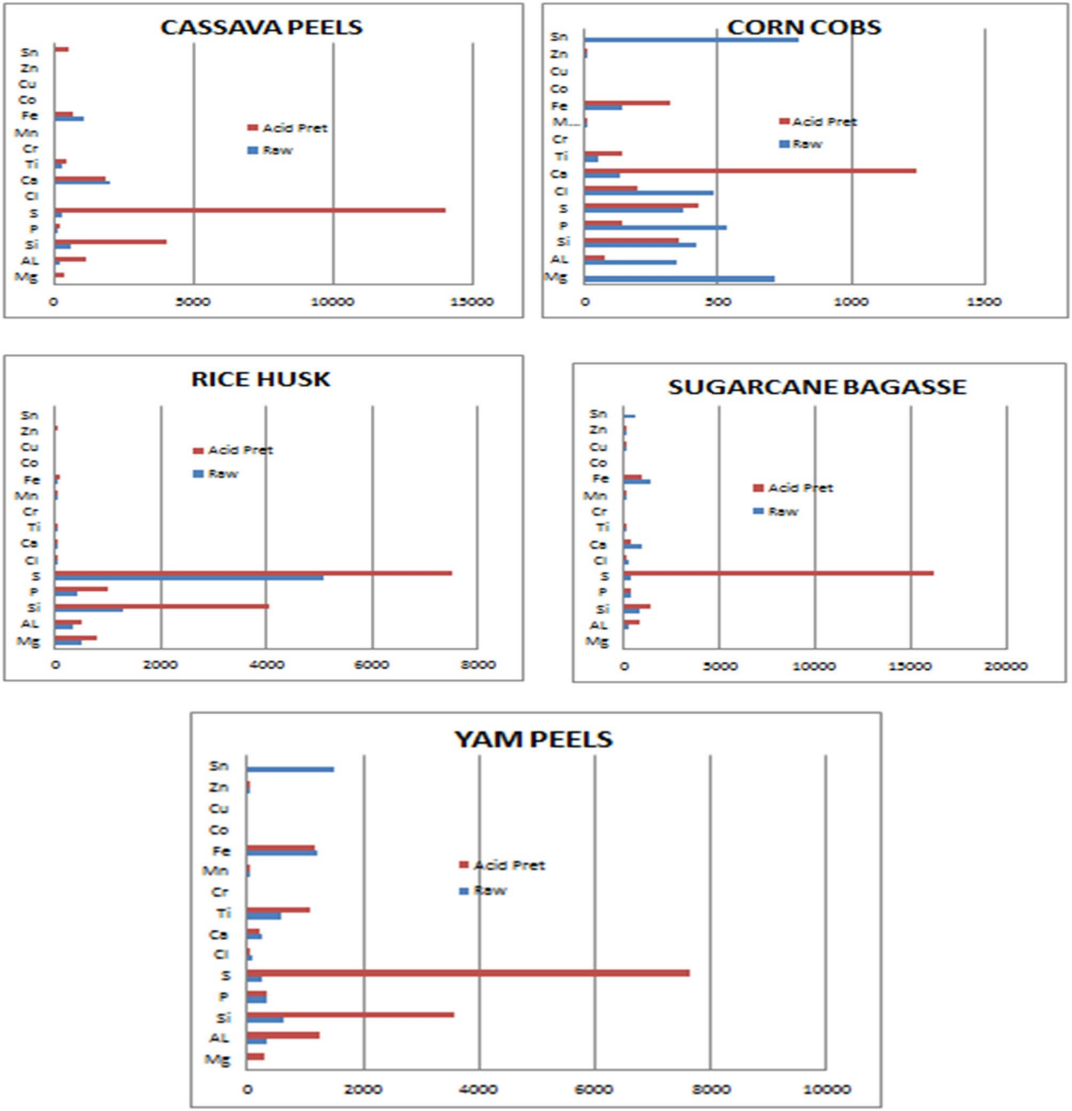
Effect of acid pretreatment for all single samples.

Table 4 and Fig. 1 show the elemental composition of the different inorganic matter present in the raw and pretreated samples of 300 μm sized particles of cassava peels, corn cobs, rice husks, sugarcane bagasse, and yam peels. From the table of results, it was seen clearly that acid pretreatment varied the elemental composition of the biomass by changing their concentration and not necessarily the elemental constituents. Nevertheless, few samples showed the presence or absence of an element before or after pretreatment. Generally, Mg, Al, Si, P, S, Ti, and Mn showed an increase in concentration across all biomass samples with exception of corn cobs for Mg, Al, Si, and P, and sugarcane bagasse for P alone. The raw sample of corn cobs contained 712 ppm of Mg whereas the acid pretreated sample contained 0.000 ppm of Mg. Also, the raw sample of yam peel biomass contained 0.000 ppm of Mg while the acid pretreated sample contained 287 ppm of Mg. The increase in the sulfur content of the acid pretreated sample against the raw sample was very high, this could be as a result of the sulfuric acid used in the acid pretreatment. There was a decrease in the concentration of Cl and Ca for all samples after acid pretreatment. Acid pretreatment could be useful in reducing the number of certain minerals present in the biomass.

**Table 4:** Effect of acid pretreatment on the raw biomass samples.

	Mg	Al	Si	P	S	Cl	Ca	Ti	Cr	Mn	Fe	Co	Cu	Zn	Sn
**Cassava peels (ppm)**															
R	55	175	630	117	287	47	2014	284	9	19	1074	0	0	18	0
A	354	1111	4014	239	14,044	18	1805	454	9	15	654	0	0	9	523
**Corn cobs (ppm)**															
R	712	344	416	535	374	487	135	53	0	11	144	0	0	13	803
A	0	76	351	142	429	198	1242	146	0	10	317	0	0	8	0
**Rice husks (ppm)**															
R	511	324	1299	431	5083	33	68	29	0	18	36	0	0	0	0
A	774	490	4070	985	7517	14	65	58	0	28	72	0	0	9	0
**Sugarcane bagasse (ppm)**															
R	0	194	824	332	348	250	901	123	0	17	1368	0	10	14	612
A	0	796	1425	311	16,197	15	382	128	0	98	955	0	34	15	0
**Yam peels (ppm)**															
R	0	331	631	334	263	74	272	589	0	61.5	1213	0	0	8	1516
A	287	1235	3574	353	7641	17	217	1085	0	33	1170	0	0	7	0

R = Raw biomass.

A = Acid pretreated biomass.

#### Alkaline pretreatment

**Figure 2:**
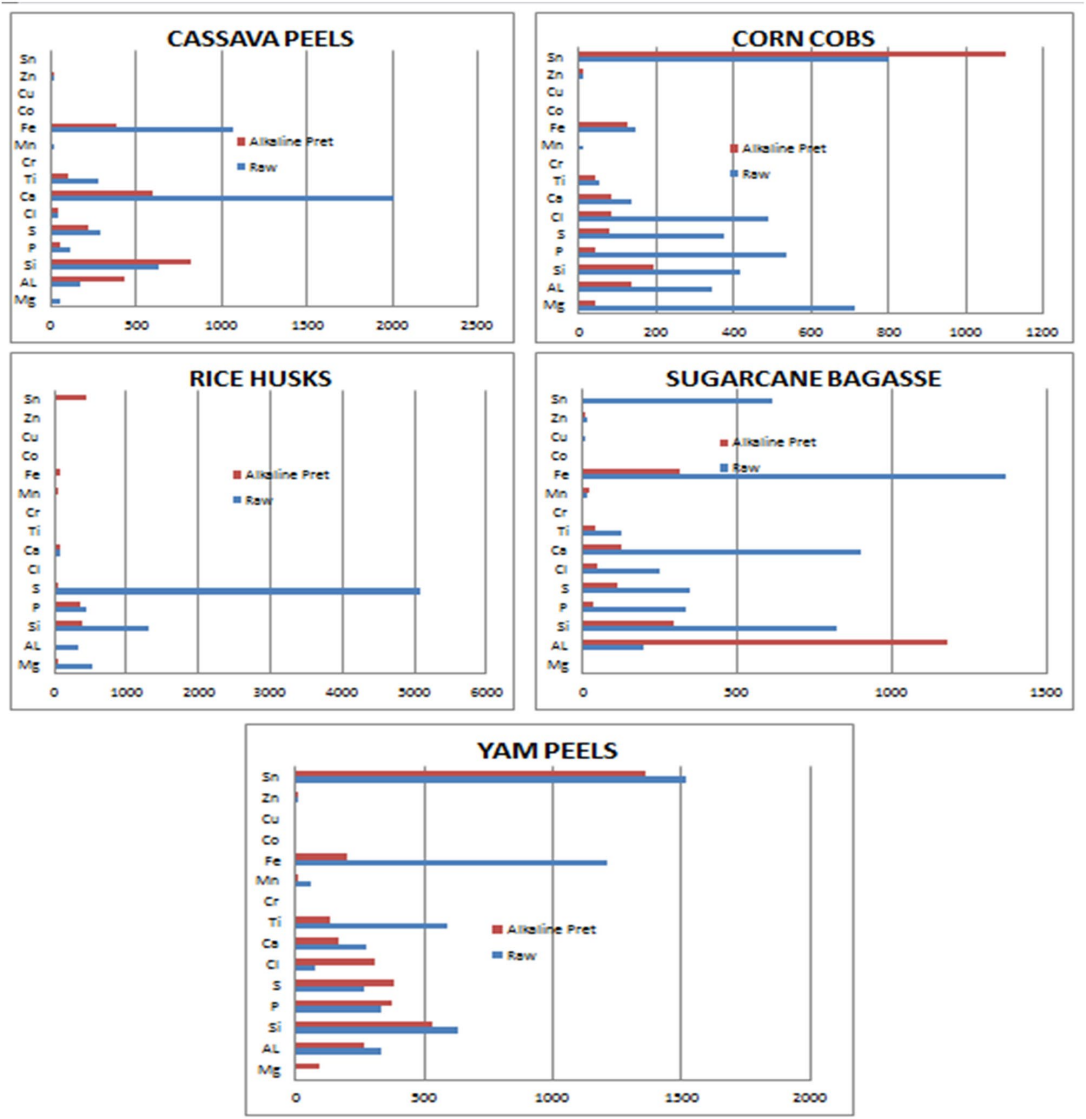
Effect of alkaline pretreatment for all single samples.

Table 5 and Fig. 2 show the results of alkaline pretreatment on the elemental composition of the different biomass. Alkaline pretreatment of the biomass samples brought about a different variation in the elemental composition of ashes from the different biomass samples. The concentration of Si and Cl in all five biomass samples decreased after alkaline pretreatment. Except for yam peels biomass, Mg, P, S, and Cl also show a decrease in concentration after pretreatment in an alkaline medium as well as Al, Cr, Mn, and Fe. Zn and Sn were seen to show a general increase in their concentration after alkaline pretreatment. The two elements were absent (showing 0.000 ppm for both) in the raw/unpretreated sample of rice husk but present after the samples were pretreated in an alkaline medium showing 18 ppm and 430 ppm respectively. The calcium concentration decreased drastically in all the biomass samples most probably because of the sodium hydroxide used for the alkaline pretreatment. Calcium is higher up the electrochemical series and for positive ions, the ones higher up the series displaces the ones lower in the series which could be the reason for the reduced calcium after the alkaline pretreatment. Another factor that could have led to the reduction of the calcium after the alkaline pretreatment could be leaching due to the high pH of the NaOH used for the pretreatment as corroborated by Osman et al. in their work^18^.

**Table 5:** Effect of alkaline pretreatment on the raw biomass samples.

	Mg	Al	Si	P	S	Cl	Ca	Ti	Cr	Mn	Fe	Co	Cu	Zn	Sn
**Cassava peels (ppm)**															
R	55	175	630	117	287	47	2014	284	9	19	1074	0	0	18	0
B	0	436	821	58	223	40	599	100	0	0	383	0	0	14	0
**Corn cobs (ppm)**															
R	712	344	416	535	374	487	135	53	0	11	144	0	0	13	803
B	41	137	194	43	81	85	84	41	0	0	124	0	0	14	1105
**Rice husks (ppm)**															
R	511	324	1299	431	5083	33	68	29	0	18	36	0	0	0	0
B	53	28	376	356	54	30	66	34	0	45	81	0	0	18	430
**Sugarcane bagasse (ppm)**															
R	0	194	824	332	348	250	901	123	0	17	1368	0	10	14	612
B	0	1180	292	35	115	48	128	39	0	24	131	0	0	9	0
**Yam peels (ppm)**															
R	0	331	631	334	263	74	272	589	0	61.5	1213	0	0	8	1516
B	92	265	534	374	385	307	168	132	0	12	201	0	0	9	1364

R—Raw biomass sample.

B—Alkaline pretreated.

#### Hydrothermal pretreatment

**Figure 3:**
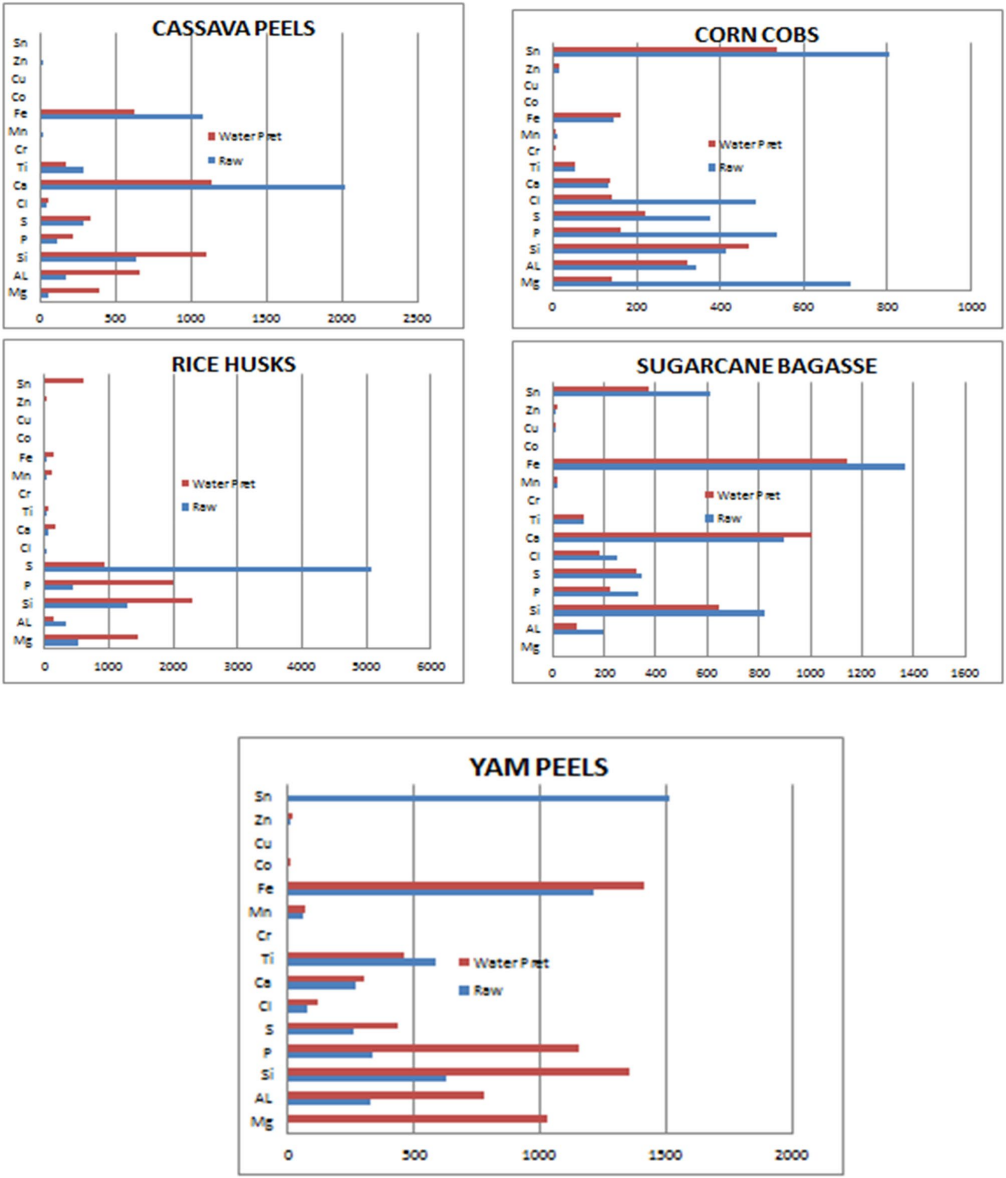
The effect of Hydrothermal pretreatment for all single samples.

Like alkaline pretreatment, hydrothermal pretreatment brought about a different variation in the elemental composition in the ash content of the five biomass samples. Except for Co and Cu, all the other samples had no clear increase or decrease in the concentration of the elements under consideration. Al, S, Cl, Ti, and Sn had a decrease in their concentration in the different samples but with exceptions in cassava peels, yam peels, for Al, S, and Cl; and corn cobs and rice husks for Ti and rice husks for Sn. Ca, Mg, Si, P, Mn, Fe, Co, Cu, and Zn all showed a general increase in their concentration in the ash content of the biomass samples after pretreatment though with exceptions. Co was 0.000 ppm in raw yam peels but 11 ppm in the hydrothermally pretreated sample of the same biomass. Table 6 and Fig. 3 show the effect of hydrothermal pretreatment.

**Table 6:** Effect of hydrothermal pretreatment on the raw biomass samples.

	Mg	Al	Si	P	S	Cl	Ca	Ti	Cr	Mn	Fe	Co	Cu	Zn	Sn
Cassava peels (ppm)															
R	55	175	630	117	287	47	2014	284	9	19	1074	0	0	18	0
C	393	664	1104	219	333	57	1136	176	0	11	628	0	0	9	0
**Corn cobs (ppm)**															
R	712	344	416	535	374	487	135	53	0	11	144	0	0	13	803
C	142	323	468	160	222	140	135	55	6	8	161	0	0	16	536
**Rice husks (ppm)**															
R	511	324	1299	431	5083	33	68	29	0	18	36	0	0	0	0
C	1461	134	2303	1986	939	0	170	58	0	103	148	0	0	32	613
**Sugarcane bagasse (ppm)**															
R	0	194	824	332	348	250	901	123	0	17	1368	0	10	14	612
C	0	92	648	220	325	179	1009	121	0	20	1141	0	14	15	376
**Yam peels (ppm)**															
R	0	331	631	334	263	74	272	589	0	61.5	1213	0	0	8	1516
C	1032	780	1356	1154	434	120	302	463	0	68	1411	11	0	15	0

R—Raw biomass sample.

C—Hydrothermal pretreated.

### Effects of mixed biomass

#### Cassava peels + Yam peels mixed biomass

**Figure 4:**
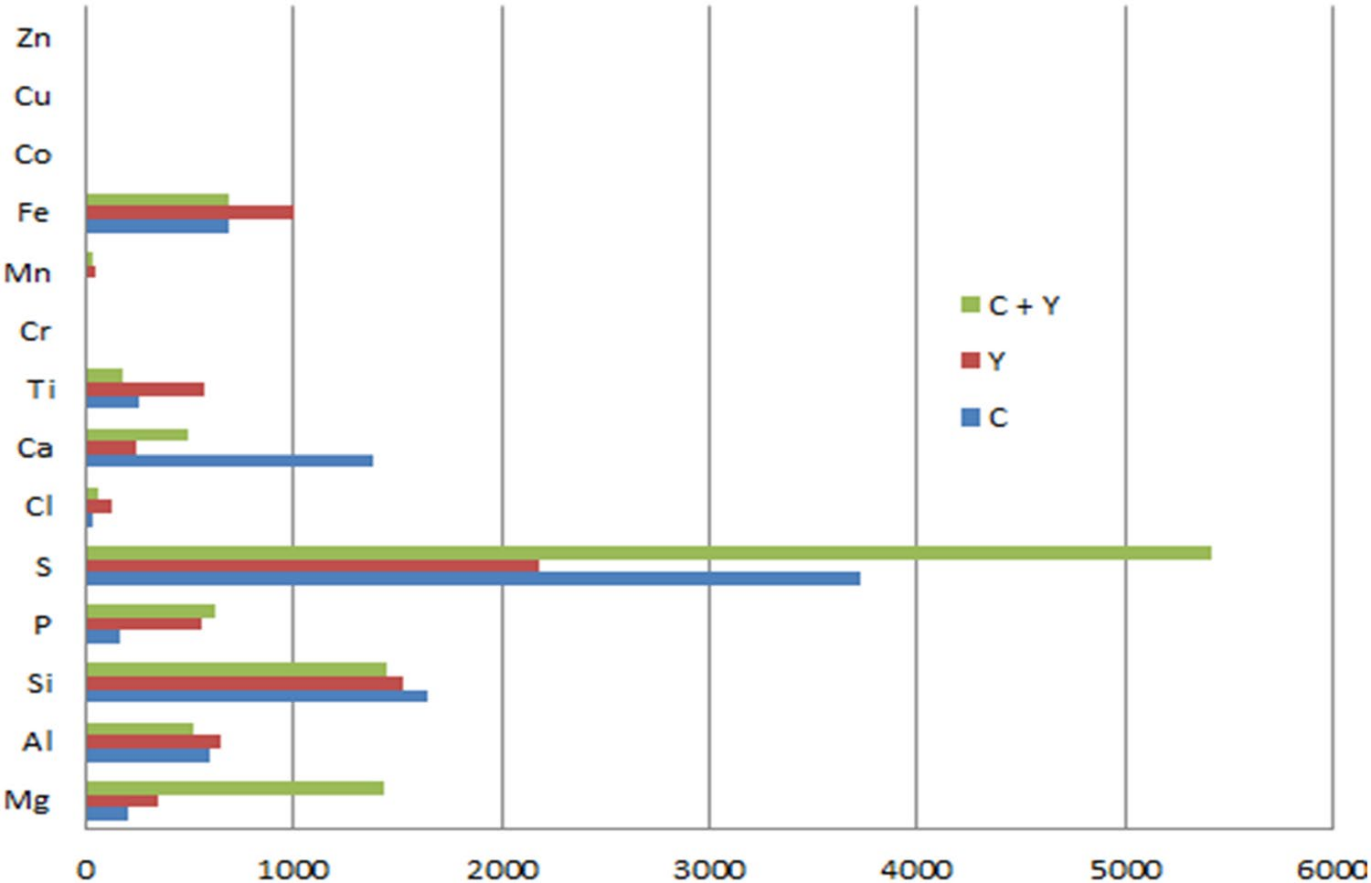
The average elemental composition of elements in cassava peels, yam peels, and cassava + yam peels.

Table 7 and Fig. 4 show the results of cassava + yam peels biomass. The pretreated samples for cassava + yam peels mixed biomass have varying concentrations of the different elements. The average Mg, P, S, and Zn concentration in the cassava + yam peels mixed biomass was seen to have increased more than they were for the individual biomass. For example, the concentration of Mg in the mixed biomass was 1441 ppm but was 200 ppm and 353 ppm in cassava and yam respectively. A decrease in concentration was however noticed in the cassava + yam peels mixed biomass for Al, Si, and Ti, when compared to the average composition of the elements in the individual cassava and yam, peels biomass. Ti in the cassava + yam peels mixed biomass was 181 ppm but was 253 ppm and 567 ppm in individual cassava and yam peels biomass respectively. The concentration of Cl, Ca, Cr, Mn, Fe, and Sn in the cassava + yam peels mixed biomass fell between the concentration of these elements in the individual cassava and yam peels biomass. As stated by Smith et al.^25^ the composition of biomass changes when two or more biomasses are combined.

**Table 7:** Average composition of elements in cassava peels, yam peels, and cassava + yam mix.

	Mg	Al	Si	P	S	Cl	Ca	Ti	Cr	Mn	Fe	Co	Cu	Zn	Sn
C (ppm)	200	597	1642	158	3722	40	1389	253	4	11	685	0	0	12	131
Y (ppm)	353	653	1524	554	2181	130	240	567	0	44	999	3	0	10	720
C + Y (ppm)	1441	523	1443	627	5421	60	492	181	3	38	685	0	3	13	648

C—Cassava peels biomass.

Y—Yam peels biomass.

C + Y—Cassava + yam peels biomass.

The choice of biomass for a given use depends on the requirements of the operation or use as each of these elements has its environmental and energy impact on the surroundings and the system. The results for magnesium, calcium and chlorine are in tandem with those obtained by Sadawi et al.^26^ in their work on commodity fuels from biomass through pretreatment and torrefaction: effects of mineral content on torrefied fuel characteristics and quality.

#### Corn cobs + Rice Husks mixed biomass

**Figure 5:**
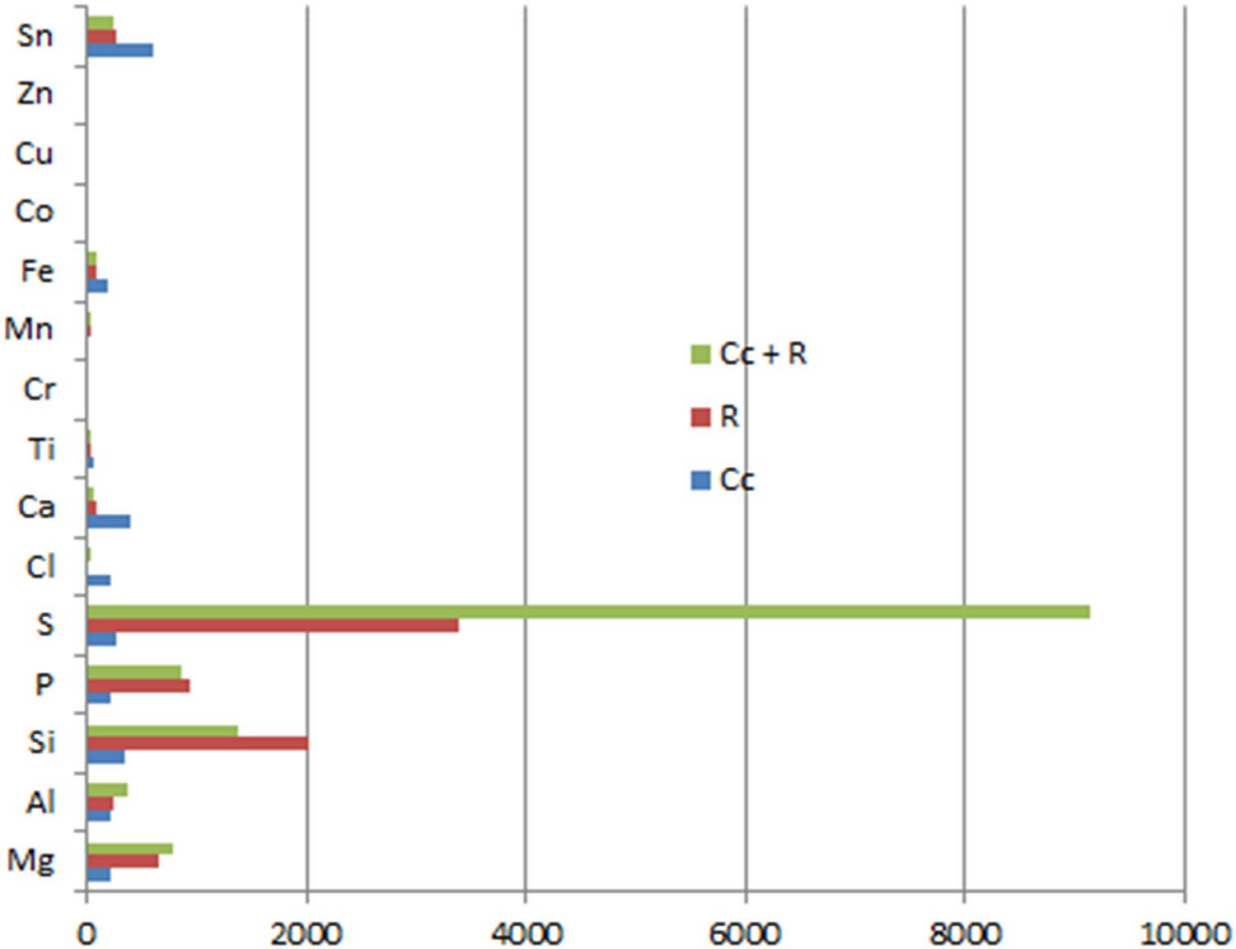
The average elemental composition of elements in Cassava Peels, Yam peels, and Cassava + Yam peels.

The pretreated corn cobs + rice husks mixed biomass has a varying concentration of elemental composition as seen in Table 8 and Fig. 5 respectively. The average Mg, S and Al concentration in the corn cobs + rice husks mixed biomass increased more than they are for the individual biomass. For example; the concentration of Mg in the mixed biomass was 800 ppm but 224 ppm and 670 ppm in individual corn cobs and rice husks respectively. Sulfur (S) in the mixed biomass was 9128 ppm but 276 ppm and 3398 ppm in individual biomass samples of corn cobs and rice husks respectively. A decrease in concentration was observed in the corn cobs + rice husks mixed biomass for Ca, and Sn when compared to the average composition of the elements in the individual biomass with 71 ppm for the mixed biomass and 399 ppm and 92 ppm for corn cobs and rice husks respectively. The other elements present in the biomass mixture took a position between the concentration of the individual biomass of corn cobs and rice husks with exception of Zn and Ti which had values equal to the least in the range. The result of the analysis shows there is a considerable change in the elemental composition of the mixture of the corn cobs and rice husks after pretreatment as compared to the singular biomass buttressing the fact that the combination of biomass feedstocks has a significant effect on their properties and subsequent output as corroborated in the previous work by the authors^14^. The high value of Phosphorus in the ash of individual and combined biomass of corn cobs and rice husks could be due to the use of fertilizers for crop cultivation^27^.

**Table 8:** Average concentration of elements in corn cobs, rice husks, and corn cobs + rice husks mixed biomass.

	Mg	Al	Si	P	S	Cl	Ca	Ti	Cr	Mn	Fe	Co	Cu	Zn	Sn
Cc	224	220	357	220	276	228	399	74	1	7	187	0	0	13	611
R	670	244	2012	940	3398	19	92	45	0	48	84	0	0	15	261
Cc + R	800	387	1370	867	9128	39	71	45	0	29	98	0	0	13	239

Cc—Corn cobs.

R—Rice husks.

Cc + R—Corn cobs + Rice husks mixed biomass.

#### Mixture of all five biomasses

**Figure 6:**
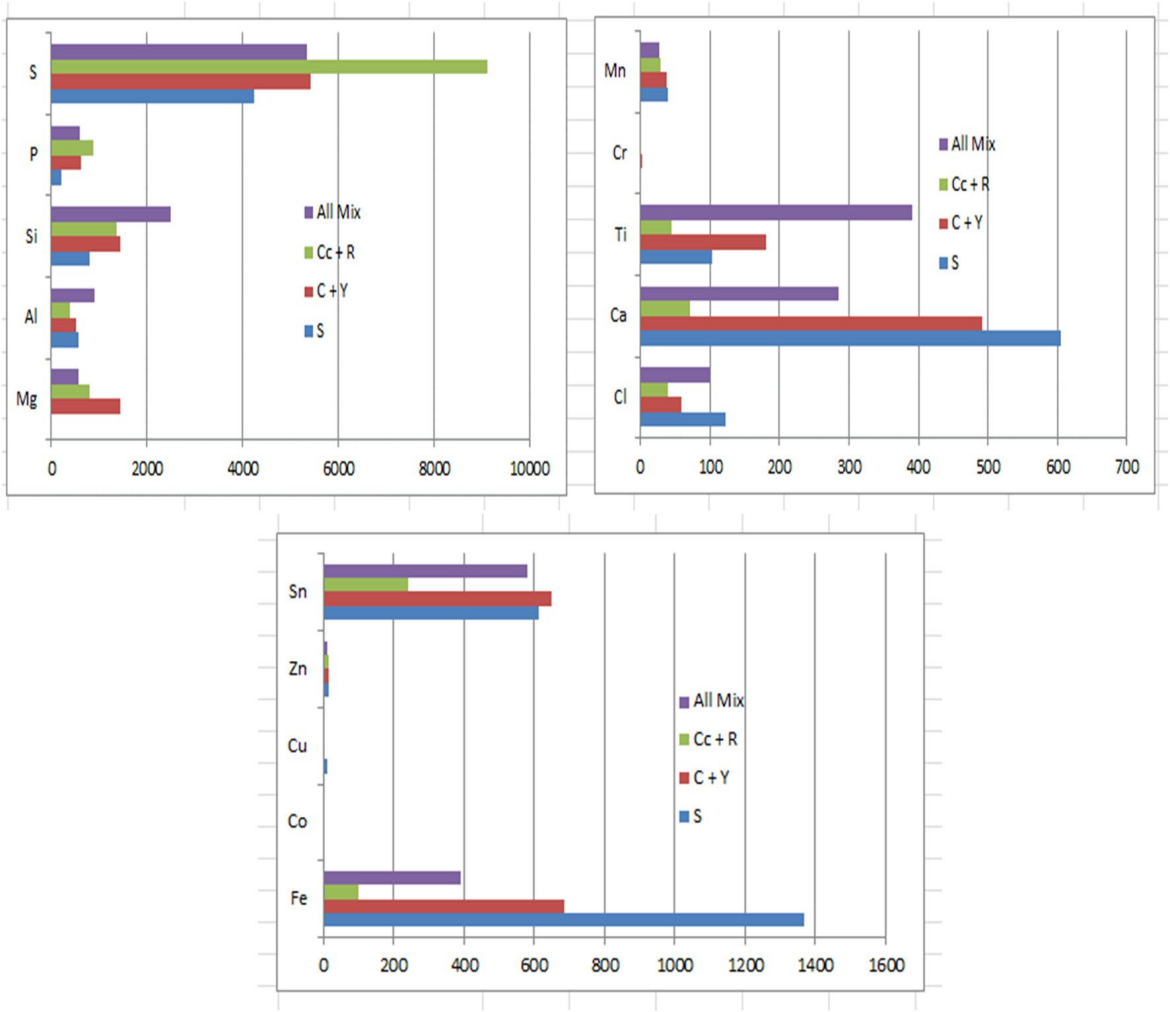
The average elemental composition in sugarcane bagasse, cassava peels + yam peels mixed biomass, corn cobs + rice husks mixed Sbiomass and the mixture of all five biomass samples.

In analyzing the impact of the mixture of all five biomass samples on the overall elemental composition of the pretreated samples, the average values of the concentration of each element in the mixture of all the five biomass are as shown in Tables 9 and 10 and also depicted in Fig. 6. From Table 10, it was seen that the concentration of Al, Si and Ti in the different biomass considered was the peak in the mixture of all the five biomass samples, with values of 909 ppm for the five biomass mixture and 565 ppm, 523 ppm, and 387 ppm for S, C + Y, and Cc + R respectively for Al. It was also evident from Table 9 that Mg (with exception of sugarcane bagasse where it was 0 ppm), Mn, and Zn had the least values for the five biomass mixtures with 28 ppm in all biomass mixture and 40 ppm, 38 ppm, and 29 ppm for S, C + Y, and Cc + R respectively. Although the difference in the concentration of Mn and Zn is insignificant as compared to the difference in the concentration of Mg.

**Table 9:** Concentration of elements in cassava peels + yam peels mixed biomass, corn cobs + rice husks mixed biomass and the mixture of all five biomass samples after pretreatment.

	Mg	Al	Si	P	S	Cl	Ca	Ti	Cr	Mn	Fe	Co	Cu	Zn	Sn
**Cassava peels + yam peels (ppm)**															
A	900	604	1021	591	1567	6	306	147	8	43	660	0	0	11	0
B	0	266	581	88	130	39	287	88	0	17	380	0	0	10	1045
C	1441	700	2726	1201	461	135	883	308	0	54	1015	0	10	18	899
Mean	780	523	1443	627	5421	60	492	181	3	38	685	0	3	13	648
**Corn cobs + rice husks (ppm)**															
A	1465	485	1856	1236	18,135	9	87	55	0	41	138	0	0	15	0
B	136	288	884	497	120	69	56	34	0	17	59	0	0	12	479
Mean	800	387	1370	867	9128	39	71	45	0	29	98	0	0	13	239
**All five biomass mixed (ppm)**															
A	596	1644	3683	313	9867	38	479	782	0	23	794	0	0	7	494
B	445	931	2502	328	5864	143	223	335	0	9	207	0	0	7	664
C	632	151	1287	1139	308	119	156	60	0	53	170	0	0	18	587
Mean	558	909	2491	593	5346	100	286	392	0	28	390	0	0	11	582

A = Acid pretreated.

B = Alkaline Pretreated.

C = Hydrothermal pretreated.

**Table 10:** Average composition of elements in sugarcane bagasse, cassava peels + yam peels mixed biomass, corn cobs + rice husks mixed biomass and the mixture of all five biomass samples.

	Mg	Al	Si	P	S	Cl	Ca	Ti	Cr	Mn	Fe	Co	Cu	Zn	Sn
S	0	565	797	224	4246	123	605	103	0	40	1368	0	10	14	612
C + Y	1441	523	1443	627	5421	60	492	181	3	38	685	0	3	13	648
Cc + R	800	387	1370	867	9128	39	71	45	0	29	98	0	0	13	239
All Mix	558	909	2491	593	5346	100	286	392	0	28	390	0	0	11	582

S = sugarcane bagasse.

C + Y = Cassava plus yam peels.

Cc + R = Corn cobs plus rice husks.

## Conclusion

The results obtained from the XRF analysis of the five lignocellulosic biomass as well as their mixed forms show that pretreatment of samples plays a significant role in modifying the elemental composition of the biomass and this varies with pretreatment type as well. Thus, the effect of acid pretreatment on a biomass sample differs from that of alkaline pretreatment on that same sample. The alkaline pretreatment on the biomass samples show better results, especially on the hybridized (mixed) feedstocks with sulfur having lower concentration than that of acid pretreatment. In the hybridized biomass, the concentration of Al, Si, and Ti in the different biomass considered was the highest in the overall mixture of all the five biomass. For Aluminum, the reported values were 909 ppm for the five-biomass mixture and 565 ppm, 523 ppm, and 387 ppm for S, C + Y, and Cc + R respectively. Information on the elemental and ash composition of biomass is vital for a bioethanol processing plant as the elemental and ash components of the biomass should be given adequate consideration during process design and the selection of process parameters because certain elements may pose a harm to the ecosystem if their concentrations are too high.
